# Spatial Scale, Means and Gradients of Hydrographic Variables Define Pelagic Seascapes of Bluefin and Bullet Tuna Spawning Distribution

**DOI:** 10.1371/journal.pone.0109338

**Published:** 2014-10-27

**Authors:** Diego Alvarez-Berastegui, Lorenzo Ciannelli, Alberto Aparicio-Gonzalez, Patricia Reglero, Manuel Hidalgo, Jose Luis López-Jurado, Joaquín Tintoré, Francisco Alemany

**Affiliations:** 1 Balearic Islands Coastal Observing and Forecasting System (SOCIB), Palma de Mallorca, Balearic Islands, Spain; 2 College of Earth, Ocean, and Atmospheric Sciences (CEOAS), Oregon State University, Corvallis, Oregon, United States of America; 3 Instituto Español de Oceanografía - Centre Oceanogràfic de les Balears (COB-IEO), Palma de Mallorca, Balearic Islands, Spain; 4 Institute for Advanced Studies (IMEDEA), Consejo Superior de Investigaciones Científicas (CSIC) and the University of the Balearic Islands (UIB). Esporles, Spain; Aristotle University of Thessaloniki, Greece

## Abstract

Seascape ecology is an emerging discipline focused on understanding how features of the marine habitat influence the spatial distribution of marine species. However, there is still a gap in the development of concepts and techniques for its application in the marine pelagic realm, where there are no clear boundaries delimitating habitats. Here we demonstrate that pelagic seascape metrics defined as a combination of hydrographic variables and their spatial gradients calculated at an appropriate spatial scale, improve our ability to model pelagic fish distribution. We apply the analysis to study the spawning locations of two tuna species: Atlantic bluefin and bullet tuna. These two species represent a gradient in life history strategies. Bluefin tuna has a large body size and is a long-distant migrant, while bullet tuna has a small body size and lives year-round in coastal waters within the Mediterranean Sea. The results show that the models performance incorporating the proposed seascape metrics increases significantly when compared with models that do not consider these metrics. This improvement is more important for Atlantic bluefin, whose spawning ecology is dependent on the local oceanographic scenario, than it is for bullet tuna, which is less influenced by the hydrographic conditions. Our study advances our understanding of how species perceive their habitat and confirms that the spatial scale at which the seascape metrics provide information is related to the spawning ecology and life history strategy of each species.

## Introduction

Seascape ecology represents an emerging field in the study of how the habitat structure shapes the spatial distribution of marine species and influences key ecological processes [1],[2]. This discipline initiated applying techniques and metrics from the traditional landscape ecology to characterize and quantify spatial structure of benthic habitats, observed as a mosaic of patches of different habitat classes [1],[3],[4], [5],[6]. Nevertheless, there is still a gap in the development of concepts and techniques providing metrics to characterize the spatial structure of the seascape in the pelagic environments, where there are no clear boundaries delimitating different habitats [1],[2],[6]. In the framework of landscape ecology spatial gradients have been recently proposed as more appropriated metric than traditional categorical patch mosaic based metrics to characterize continuous habitats [7]. Accordingly, a location in a pelagic seascape would be better characterized by the combination of the value of a particular hydrographic variable and its spatial gradient.

Several studies have applied gradients of hydrographic parameters to characterize the spatial distribution of marine species during various life history stages, as nursery, foraging or spawning [8],[9],[10],[11],[12]. It is likely that the scale at which an individual perceive a change in the environment (i.e., a gradient) varies according to life history, ontogeny and to the hydrographic variable in exam. For instance, while large-scale gradients associated with the North Pacific transition zone drive the location of many pelagic predators including albacore tuna (*Thunnus alalunga*) during their feeding migratory stages [13], once off the west coast of the US, albacore tuna distribution is associated with smaller scale features linked to upwelling fronts [14]. In the Mediterranean Sea during spawning, bluefin tuna distribution is regulated by oceanographic variables that can change at relatively small scales [15],[16]. In spite of the expected importance of gradient scales, to our knowledge there are no studies that have evaluated the effect of changing the spatial scales at which environmental gradients are calculated to model the spatial distribution of fish. The goal of our study is to examine the distribution of large pelagic predators during spawning by explicitly considering mean, gradients and scale of gradients of hydrographic variables.

**Figure 1:**
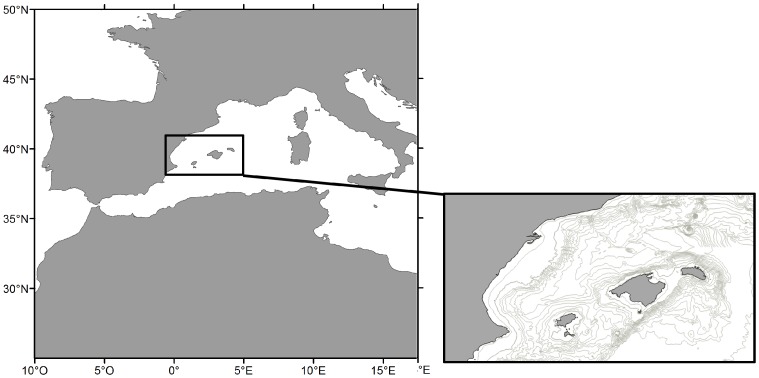
Location of the Balearic Islands, Western Mediterranean.

Atlantic bluefin tuna (*Thunnus thynnus*) and bullet tuna (*Auxis rochei rochei*) are two species of pelagic predators showing different spawning strategies. We target these species in the Balearic Sea (Figure 1), known as a recurrent spawning area for large pelagic species in the Western Mediterranean [17]. Bluefin tuna is a highly migratory species; the Eastern population enters in the Mediterranean Sea from the North Atlantic at the end of spring and early summer [18],[19]. Their spawning activity at the Balearic Sea is linked to the regional oceanography with spawning grounds located in the vicinity of frontal structures formed when the recent Atlantic water mass encounters the more saline resident surface Atlantic waters [15]. The area is characterized by highly dynamic processes that promote a seascape shaped by filaments, fronts and eddies whose location varies between years [20],[21]. Bullet tuna, by contrast, is smaller and more frequent in near coastal areas [22]. The spawning of bullet tuna is associated with the geography. Young larvae are found mainly in coastal areas and with little influence of the local oceanography in comparison with bluefin [16].

We expect that, when selecting spawning locations, a large-bodied and long-distance migratory pelagic fish, such as the bluefin tuna explores its environment at larger spatial scales than bullet tuna, a small-bodied and non-migratory pelagic fish. Therefore, we expect that pelagic seascape metrics based on the combination of hydrographic parameter values and their gradients calculated at appropriate spatial scales provide relevant information for bluefin tuna but not for bullet tuna, where we expect a greater reliance on geographic and hydrographic parameters, calculated at comparatively small-scale.

**Figure 2:**
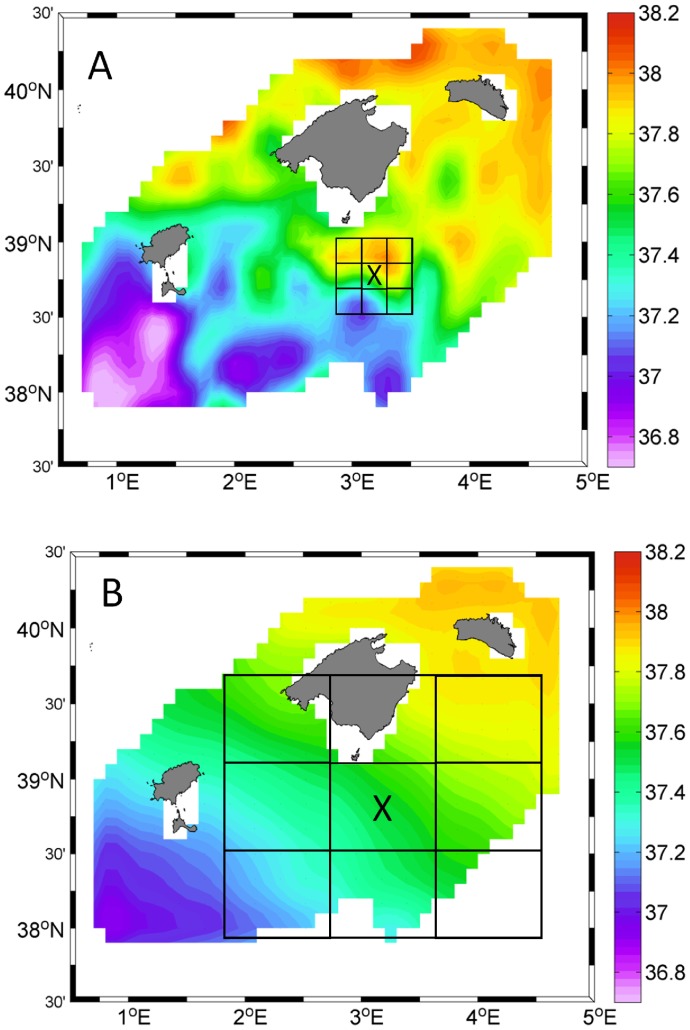
Sea surface salinity field in 2003. Spatial means of sea surface salinity processed at two different scales A) 0.15 degrees and B) 0.75 degrees. Spatial means were interpolated following an objective analysis onto a regular grid by using minimum error variance methods (Bretherton, 1976). The squares in each figure show the polygons used for the calculation of the spatial gradients at the two scales at station X.

In this work we analyze the influence of the pelagic environment by depicting the spatial scales at which gradients of hydrographic variables are linked to the spawning ecology of these tuna species. We investigate the two most relevant hydrographic variables describing their spawning spatial distribution: salinity and geostrophic currents velocity [16], already determined in previous studies. Our analytical approach has two steps. Firstly, we identify the scale at which each hydrographic variable (Figure 2) maximizes the performance of a model fitted to larval distribution. Second, we evaluate whether components of the seascape (i.e., mean and gradients of oceanographic variables) are interactively affecting the spatial distribution of tuna larvae. By performing this analysis on two species we evaluate how fish with contrasting life history strategies perceive their environments when deciding for spawning locations.

## Materials and Methods

### Data acquisition

Bluefin and bullet tuna larvae were collected during ichthyoplankton surveys using Bongo nets from 2001 to 2005. The surveys were conducted by the Instituto Español de Oceanografía (www.ieo.es), a Spanish Government marine research institution. The sampling scheme was communicated and approved by the Spanish Directorate of Fisheries before the sampling was conducted. No specific ethical approval was required and the survey of biological data was conducted using Bongo nets,which are accepted standard techniques for this type of surveys, used worldwide for the collection of plankton samples, including billfish and tuna larvae [15],[17], [23], [24]. The nets were towed at low speeds, around 2 knots, during 8–10 minutes, and plankton samples were immediately preserved with 4% formalin buffered with borax onboard.

Around 200 stations were sampled every year, in a regular sampling grid of 10×10 nm located between 37.85°–40.35° N and 0.77°–4.91°E, covering a total area of 101360 km2 ( = 280×362 km) around the Balearic archipelago. The sampling was conducted during June–July coinciding with the spawning period of bullet and bluefin tuna (see [15] for details of the sampling procedures). Tuna larvae were identified to the species level and measured in standard length. All larvae identified as yolk sac and preflexion stages (<4.5 mm) were classified as “stage 1”.

Mean and maximum age of larvae under this size are 6 days and 11 days old respectively, accounting for growth [25] and hatching time [26]. Displacement from spawning areas during these intervals in the study area are below 25 kilometers for the mean ages, which are in the range of 1.4 sampling stations, and 46 kilometers for the maximum ages, what is in the range of 2.6 sampling stations. These values have been calculated following methods in [27]. Considering these values, abundances of stage 1 larvae have been defined to get a proxy of spawning locations as in previous research in the study area [28].

Vertical profiles of conductivity, temperature and pressure data were recorded at all stations, by means of Sbe911 CTD. Sea surface salinity at each station (SAL) was calculated as the mean salinity over the mixed layer depth. Geostrophic velocities (GVEL) were calculated at sea surface from the first-derivative of the sea surface height between adjacent points, which was obtained by vertical integration of the specific volume, using 600 m as the level of no motion [21].

These two variables (SAL and GVEL) were selected, since they have been demonstrated to be the two most relevant environmental variables describing the spawning spatial distribution of tuna [16]. Sea surface temperature was also included in the models since in this area it is a secondary but relevant variable mainly related to the phenology of the spawning process [29]. However, the spatial gradient was not explored because sea surface temperature during the summer changes relatively fast due to solar irradiance [21].

### Processing of spatial gradients along continuous spatial scales

Spatial gradients from the sea surface salinity field (gSAL) and geostrophic velocity field (gGVEL) within the sampled region were calculated at six spatial scales, from 0.15° to 0.90° with a spatial increment of 0.15°. The minimum (0.15°) and the maximum scale (0.90°) were in the range of the smallest (from 0.13° to 0.27°) and largest (up to 0.92°) mesoscale oceanographic structures in the area [21]. For the computation of the gradients, nine square polygons at every scale were delimitated around each sampling position (see examples for scale 0.15° and 0.75° in figure 2A, 2B respectively). The gradient was then computed as the maximum absolute difference between the mean hydrographic variable at the center polygon and each of the eight surrounding polygons standardized to distance [9]. The software for the spatial processing was developed in R language [30].

### Identification of spatial scales

Comparison of how models perform along scales allowed identifying the spatial scales at which information provided by gradients is maximized. The effect of gGVEL and gSAL at each scale on the abundance of bullet and bluefin tuna larvae was assessed using nonparametric regression statistical models (generalized additive models, GAMs, [31]). A base model was formulated to describe inter-annual variability (variable YEAR), sampling location (latitude and longitude variables), and the hour of the day on the catch of tuna larvae. Over-dispersed Poisson distribution family and a natural-log link were selected to model larval data. The volume of water filtered was included as an offset (after natural log transformation), to account for the effort expanded in catching the sample (Equation 1). The effects of these variables on the base model have been already analyzed in previous studies [16]. Here, the base model represented the null hypothesis of no gradient effect on tuna larvae distribution, against which all other more complex formulations will be compared.

Equation 1: Base model
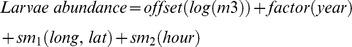
m3 = volume filtered by the bongo nets (m3); long = longitude; lat = latitude; hour = hour of the day expressed from 0 to 1, sm_1_ and sm_2_ the smoothing functions.

At each spatial scale a GAM model was processed including the gradient of one hydrographic variable (gSAL, gGVEL) as a new additive term (s3) in the standardization model. The number of knots for the new smoother was always set to a maximum of three (i.e. two degrees of freedom) in order to avoid over fitting in the responses.

The identification of characteristic spatial scales (cgSAL, cgGVEL) was assessed with scalogram where the scale of the covariate is plotted against a measure of the model goodness of fit, which in our case were represented by the adjusted R-squared (Rsq, the higher the better), and the Generalized Cross Validation (GCV, the lower the better) [31]. We selected the scale that maximize Rsq and minimize the GCV. Results of the base model (when a seascape covariate was not included) were presented in the same graphics. Note that due to the greater complexity of the gradient model higher Rsq values do not necessarily imply an improvement in relation to the base model, while they do represent a better performance when compared to other gradient models.

Significant differences of Rsq values between models, or GCVs, were obtained from t-test of these parameters obtained from 500 iterations where 10% of the data was excluded. For all cases, alternative hypothesis (difference in means is not equal to 0) was accepted only if the P value was lower than 0.001, with a confidence level of 0.99. When one variable presented similar Rsq and GCV values at various scales, selection was assessed by inspection of the plot showing the response of the abundance in relation to the gradient processed at those scales.

Once the characteristic scale of the gradients was identified, we tackled the questions of whether the information provided by the gradients is different and complementary to the information provided by the hydrographic variables from which they were calculated, and in that case, how the information from these two variables (spatial mean and gradient) should be combined to maximize the goodness of fit of the models and the ecological information they provide. To assess these questions we analyzed the performance of models with different complexity:

- The base model from equation 1.
- Hydrographic models combining the sea surface salinity, geostrophic velocity and sea surface temperature at the sampling station (stSAL, stGVEL, stSST).
- Seascape models combining the gradients at characteristic scales (cgSAL, cgGVEL) and the hydrographic variable at the sampling location (stSAL, stGVEL, stSST). Different seascape models were constructed including the two components of the seascape (values at stations and gradients) as additive and interactive terms. An interaction may be ecologically meaningful when a species is selecting its spawning habitat on a specific side of a frontal region, for example. In such case, it is the combination of both the gradient and the mean that provide the suitable conditions for spawning. The performance of different model configurations for each species was assessed by the delta AIC (ΔAIC), calculated as the difference between model AICs and the base model AIC. The AIC in this case is best suited for model comparisons because each model had different number of variables [32]. Rsq, GCV and explained deviances were used to compare how models perform between the two species, as AIC values among models with different dependent variables are not comparable.

## Results

### Identification of characteristic spatial scales

In all years considered, the recent Atlantic water masses encountered the more saline resident water masses forming an oceanic frontal zone inside the study area. The size of such frontal zone was bigger than other oceanographic phenomena as small eddies and meanders derived from the instabilities along the haline front and the effect of strong bathymetric changes (Figures S3, S4).

**Figure 3:**
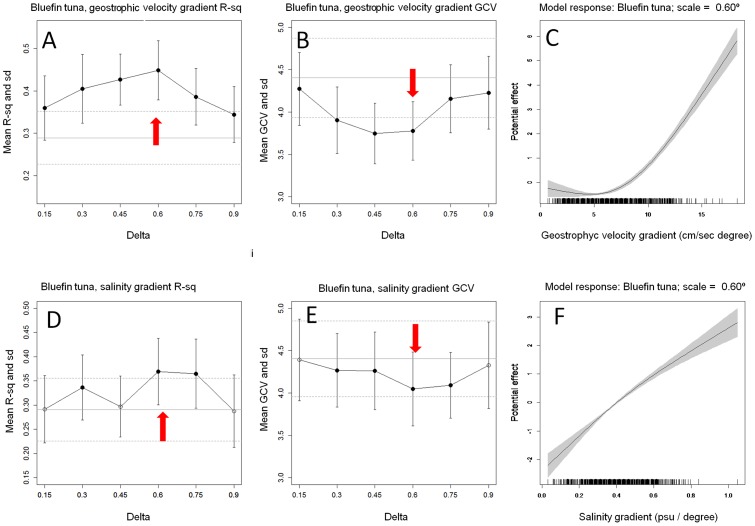
Rsq and GCV Scalograms of bluefin tuna larva abundance models along spatial scales, standard deviations. Horizontal grey lines indicate statistics from the base model (Straight line = mean, dashed line = Sd). Black dots show scales at which values are significantly different from the standardization model. Red arrows indicate the selected characteristic scale.

The scalogram of gGVEL for bluefin showed that Rsq values gradually improve as the spatial scales increased to a maximum at 0.6° (Rsq = 0.44, Figure 3A), which was chosen as the geostrophic velocity gradient characteristic scale for bluefin tuna. Values of GCV showed a similar pattern of model improvement, being significantly better than the base model at 0.6 degrees (Figure 3B). At this characteristic scale the response of the larvae abundance is positively related to the gradient of geostrophic velocity (Figure 3C).

The scalograms of gSAL for bluefin tuna showed also an increment of Rsq with higher values at 0.6° and 0.75° that also coincide with lower values of GCV (Figure 3D, 3E). Differences of R-sq between these two scales (0.6° and 0.75°) were not significant. The characteristic scale for gSAL was chosen at 0.6° as the model response at this scale presented a less ambiguous effect on larval abundance (Figure 3F). The gSAL at 0.75° spatial gradients displays a dome-shaped response with a less clear ecological interpretation (Figure S1). At this scale GCV was lower than the base model.

**Figure 4:**
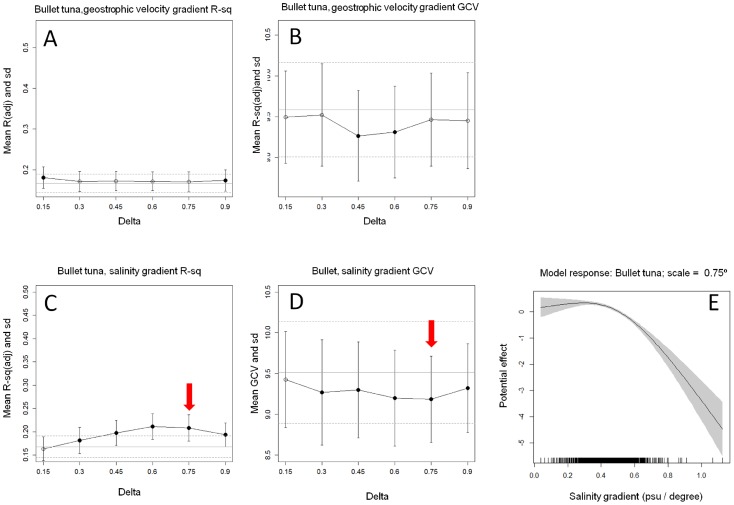
Rsq and GCV scalograms of bullet tuna larva abundance models along spatial scales, standard deviations. Horizontal grey lines indicate statistics from the standardization model (Strait line = mean, dashed line = Sd). Black dots show scales at which values are significantly different from the standardization model. Red arrows indicate selected characteristic scale.

In contrast to the results obtained for salinity, the gradients of geostrophic velocities (gGVEL) did not show any single scale that maximizes R-square and minimizes GCV for bullet tuna (Figure 4A, 4B). The Rsq scalogram showed a flat trend with the highest value at 0.15 degrees. The Rsq value at this scale ( = 0.18) showed similar values than other scales (values between 0.170 and 0.173) or when compared to the base model ( = 0.166). On the contrary the GCV scalogram showed significantly lower values than the base model at 0.45 and 0.6 degrees, scales at which Rsq were not even significantly higher than the base model. Therefore, the contradictory response of the model performance indicators, the flat trend of Rsq scalogram and their very low values (despite the higher complexity of the gradient models in relation to the base model), may indicate that the spatial gradient of geostrophic velocity is not a valid seascape metric for the spawning locations of bullet tuna. Consequently, gGVEL was excluded from further analysis in relation to this species.

The gSAL scalogram of bullet tuna showed a moderate effect of the scale at which gradients were calculated. The shape of the scalogram did not show a peak scale at which model performs better (Figure 4C). Scales from 0.45°, 0.6° and 0.75° for gSAL seemed to maximize Rsq, but not being different from each other. In this case GCV-scalograms showed the lowest at 0.75° (Figure 4D), being significantly lower than the base model, which was selected as characteristic scale. The Rsq at this scale was higher ( = 0.21) than that of the base model (Rsq = 0.16). At this scale, gradients displayed a negative effect on the bullet tuna larvae abundance (Figure 4E) showing that bullet tuna spawning locations are found with more probability in areas where salinity is spatially homogeneous– a result that contrasted to that obtained for bluefin tuna.

### Species-specific seascape characterization

The best model for each species included a gradient and a mean term (Tables 1 and 2). Note however that the hydrographic model already represents an improvement respect to the standardization model (Tables 1 and 2). For bluefin tuna the best seascape model had an improvement of 186% of the Rsq when compared to the base model (Rsq base mode = 0.23; Rsq best seascape model = 0.66, Table 1). The improvement for bullet was 61%, considerably lower compared to bluefin (Rsq base mode = 0.16; Rsq best seascape model = 0.25, Table 2).

**Table 1:** Summary of GAM models of larvae abundance for Atlantic bluefin tuna (*Thunnus thynnus*).

Model group	Model variables	R2	Dev(%)	GCV	AIC	delta AIC
Base model	(latitude, longitude) + filtered volume + hour	0.232	40.8	4,596	3985,54	0
One additive variable models	base model + stGVEL0.15	0,271	43,4	4,412	3827,79	157,75
	base model + gGVEL0.6	0,39	49,4	3,947	3465,03	520,50
	base model + stSAL0.15	0,222	41,8	4,534	3924,70	60,84
	base model + gSAL0.6	0,301	45,1	4,275	3723,53	262,01
Hydrographic model	base model + stGVEL + stSAL + stTEMP	0,472	51,6	3,814	3338,62	646,92
GVEL seascape models	Hydrographic model + stGVEL + gGVEL0.6	0,53	55,8	3,500	3087,14	898,40
	Hydrographic model + (stGVEL,gGVEL0.6)	0,666	59,3	3,251	2881,29	1104,25
SAL seascape models	Hydrographic model + stSAL + gSAL0.6	0,506	54,8	3,571	3145,80	839,74
	Hydrographic model + (stSAL,gSAL0.6)	0,533	57,1	3,417	3009,58	975,96

Interaction terms included in parenthesis.

**Table 2:** Summary of GAM models of larvae abundance for bullet tuna (*Auxis rochei rochei*). Interaction terms included in parenthesis.

Model group	Model variables	R2	Dev(%)	GCV	AIC	delta AIC
Base model	(latitude, longitude) + filtered volume + hour	0,158	32,8	9,615	8857,17	0
One additive variable models	base model + GVEL st0.15	0,177	35,3	9,281	8572,91	284,26
	base model + stSAL0.15	0,16	36,5	9,130	8440,06	417,11
	base model + gSAL0.75	0,207	39,6	8,741	8098,28	758,89
Hydrographic model	base model + stGVEL + stSAL + stTEMP	0,192	38,8	8,847	8183,39	673,78
SAL seascape models	Hydrographic model + stSAL + gSAL0.75	0,215	40,6	8,617	7984,18	872,99
	Hydrographic model + (stSAL,gSAL0.75)	0,255	43,1	8,327	7708,82	1148,35

**Figure 5:**
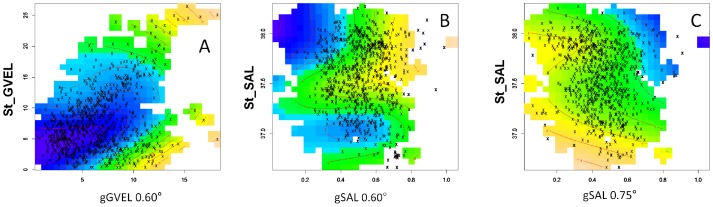
The effect of the interactions of the seascape components on the larval abundance as estimated from the seascape generalized additive model. The effects are shown for bluefin tuna (A–B) and bullet tuna (C). For bluefin tuna: A) the effect of the gGVEL and st_GVEL interaction. B) the effect f the gSAL and st_SAL interaction. For bullet tuna C) the effect of the gSAL and st_SAL interaction. Isolines indicate larval abundances predicted by the model. Peak of abundances are indicated in pink-yellow. Low and very low abundances are indicated in green and blue, respectively.

Pearson correlation coefficients between hydrographical variables and their gradients at characteristic scales were in all cases below 0.50 and pair plots showed no clear tendencies on the correlations (Figure S2), indicating that selected gradients provided complementary information to that of the hydrographical variable. Models showed a better performance (i.e. lower GCV and higher ΔAIC; Tables 1 and 2) in all the cases when the gradient and the hydrographic value were considered as an interaction term, suggesting dependence in their effect on larvae abundance rather than an additive response. However, larvae abundance of each species responded differently to the interaction of seascape components (Figures 5A, 5B, 5C).

For Bluefin tuna, higher probability of spawning was associated to higher values of geostrophic velocity gradients, but where velocities at station may present either high or low values (Figure 5A), (Figures S7, S8). Considering that a gradient is characterized by an area with high current speed near an area of low current speed, this result indicates that spawning locations were not associated to a particular side of the gradient, but in an area around the location where maximum velocities occurs. The extension of this area would be around a circle of 0.6 degrees of radius (aprox. 65 km in the study area), the characteristic scale at which the gradients were more relevant. In contrast, the interaction of the salinity seascape variables showed high larvae abundances in areas with high salinity gradients and intermediate-high salinity levels (Figure 5B), indicating an effect of the location of the main haline front and a preference for spawning at the high salinity values of that front (Figures S5, S6). The characteristic spatial correlation scale of local oceanographic structures in the area is around 18 nmi (aprox. 0.15 meridian degrees) [21] and therefore surface oceanographic structures at smaller spatial scales are ephemeral. The oceanographic structures at larger spatial scales, relevant for bluefin tuna, are linked to the Med-Atlantic salinity front and last longer.

The functional form of the interaction terms was different for bullet tuna. The relation between the bullet tuna larvae abundance and the interaction of spatial distribution of sea surface salinity and its gradients is presented in Figure 5C. Spawning locations were associated to areas where the salinity at the station were lower and gradients presented intermediate values, but the interaction plot revealed that spawning also appears in areas of higher salinities associated to very low gradients. Areas defined by this twofold combination were located at both sides of the front avoiding more mixed waters. This spatial distribution was more evident in years 2001, 2003 and 2005 (Figures S9, S10)

## Discussion

We have found that the combination of sea surface current velocities, salinities and their gradients calculated at characteristic spatial scales are relevant for the parameterization of the pelagic seascape affecting a key ecological process of bluefin tuna. For bullet tuna only salinity and their gradient provided a valid seascape metric not being relevant the gradients of sea surface current velocities. In agreement with our expectations, the importance of these metrics was much higher for bluefin, a large-bodied, long distance migratory and more dependent on local oceanography than were for bullet tuna a smaller coastal species with shorter migration distance.

Previous studies have documented the links between the frontal activity and the spawning of bluefin tuna [15],[16],[33]. In this study we add to these results by examining the effect of gradients and their interactions with hydrographic mean. These metrics improved our understanding of the conditions for bluefin and bullet tuna spawning when compared to models using just the hydrographical values but not the gradients. Furthermore the identification of characteristic scales of gradients provided a new source of information for the interpretation of how local oceanography determines the selection of the site to spawn.

For bluefin tuna larvae, spatial salinity and geostrophic velocity gradients maximize spatial model performance when calculated at 0.6 degrees. The higher abundance of bluefin tuna larvae in areas with intermediate to high salinities and with high gradients of velocity is consistent among years. Higher abundance occurs around the location of the main frontal area, at the side of higher salinity of the front and where current speed presents high values. Higher salinity water likely has higher resident time near the islands than the less saline water, which may run along the front towards east getting farther from the archipelago. Spawning at the higher salinity side may favor spatial overlap with other larval species that are also located in this water mass [17],[34].

Results for bullet tuna showed that pelagic seascape metrics are not as relevant to explain the spawning distribution as they are for bluefin. In the western Mediterranean, bullet tuna spawning has been associated to near coastal areas [22], being less influenced by the local oceanography than bluefin tuna [16], which is consistent with our results.

Despite the lower importance seascape metrics in bullet tuna, the inclusion of salinity gradients provided additional information for the identification of spawning sites. The analysis indicated that bullet tuna spawning areas are mostly found in areas where salinity gradients are low. Bullet tuna was found at both sides of the front but avoiding more mixed waters, located closer to the front. This was verified when observing the spatial distribution of larvae in relation to the salinity seascapes among the different years. For instance, in 2001, 2003 and 2005 high larvae abundances were observed North of the archipelago (high salinity waters with very low gradients), but intermediate abundances, indicating spawning, also occurs in Southern areas (low salinities and intermediate gradients). In 2002 and 2004 higher abundances were linked to low salinity and intermediate gradients shown in the south of the archipelago (Fig. S9 and S10). These results reinforce the theory of bullet tuna spawning occurs in widespread geographic areas, and not only close to the coast and suggest that the location of the main haline front negatively affects the spawning of this species.

Overall results related to bullet tuna point to the fact that, besides the avoidance of areas near strong surface haline gradients, other factors not considered in this study may also be relevant for spawning site selection in this species. It is also relevant that the spatial pattern in relation to the salinity is the opposite to that shown by bluefin tuna, located in areas near the front, suggesting possible avoidance of predators by bullet spawners [35].

The application of seascape metrics derived from salinity and geostrophic currents to characterize the spawning habitat provides new descriptors for environmental variables that improve model quality and predictions. This improvement allows a more precise identification of the relationships between the spatial location of the spawning grounds and the local oceanographic processes. Moreover, our study demonstrates that seascapes must be characterized at specific spatial scales to provide useful information as proposed in previous studies [36] and supporting results on terrestrial landscapes [37],[38] and bottom seascapes [39]. Therefore, the relations between the location of spawning sites and the mesoscale oceanographic processes may prove to be non significant if seascape metrics are not processed at the right spatial scales.

Seascape ecology is an emerging field generally being applied for the analysis of how benthic habitats pattern in coastal areas drives different aspects of marine species ecology [39]. Techniques are applied following categorical approach where the seascape is composed by a number of patches of different type of habitats [40],[7]. However, very little attention has been paid to the techniques and concepts to investigate pelagic seascape ecology due to the complex spatiotemporal dynamics of this system [2]. Thus, the work presented here sheds new light to modeling spatial distribution and investigating key ecological processes of species highly dependent on the variability of the pelagic environment, like spawning ecology of many of the big tuna species are [41]. In areas as the Balearic Sea, for which new operational oceanography platforms provide near real time data of hydrography [42] and also in combination with remote sensing data (e.g. altimetry [43]) and modeling [44],[45] these metrics will improve the species spatial distribution forecast that has proved effective for management [46].

In contrast to seascapes, landscape metrics have a long history in terrestrial ecology, and have improved over time. For instance, the effect caused on the habitat analysis derived from the spatial definition of the input habitat maps or the extent of the study area are common studied topics, [47],[48]. Likewise, calculation of seascape metrics and the final results from their application in ecological studies may be affected by different issues, like the different ways of computing the hydrographic variables and their gradients, or the origin of the input data source like from in situ measurements, remote sensing or hydrodynamic models, each with different sources of uncertainty. A relevant question is how seascapes can provide information for other type of species and ecological processes. Addressing all these challenges and developing comparative studies between different data sources, processing methods, species and ecological processes will allow advancing towards the understanding of how seascape metrics can provide information about how ecological processes and oceanography are linked together.

In summary, pelagic seascapes based on gradients and characteristic scales allow improving spatial distribution models and the identification of essential fish habitat of pelagic species. They also provide a tool for analyzing the links between particular ecological processes and local oceanography going far beyond than stochastic models based on just hydrographic parameters as salinity, temperature or geostrophic velocities. As a consequence these metrics will provide an improvement in all the management approaches and tools pending on the capability of models to identify essential habitats as near real-time spatial management based on habitat predictions [46],[10], pelagic species distribution from deterministic models [49] or the standardization of larvae indices to assess adult stock, [50],[51],[52].

## Supporting Information

Figure S1 **Model response of bluefin tuna in relation to salinity gradient processed at 0.75 degrees.** Fitted line (solid line) and 95% confidence intervals (grey shaded areas) are shown. Whiskers on the x-axis show the locations of measurements.
(TIF)

Figure S2 **Correlation between the gradients at the characteristic scales and the hydrographical variables at the sampled station.** A) Current velocity and B) salinity for Atlantic Bluefin tuna. C) Salinity for Bullet tuna.
(TIF)

Figure S3 **Sea surface salinities in the area during the five years analyzed (2001 to 2005).**
(TIF)

Figure S4 **Sea surface geostrophic currents in the area during the five years analyzed (2001–2005).**
(TIF)

Figure S5 **Spatial distribution of bluefin tuna (** ***Thunnus thynnus*** **) larvae in relation to the salinity mean calculated at its characteristic scale (0.6 degrees).** Relative stage-1 larval abundances are shown in the maps such as dots.
(TIF)

Figure S6 **Spatial distribution of bluefin tuna (** ***Thunnus thynnus*** **) larvae in relation to the salinity gradient calculated at 0.6 degrees.** Relative stage-1 larval abundances are shown in the maps such as dots.
(TIF)

Figure S7 **Spatial distribution of bluefin tuna (** ***Thunnus thynnus*** **) larvae in relation to the geostrophic velocity mean calculated at its characteristic scale (0.6 degrees).** Relative stage-1 larval abundances are shown in the maps such as dots.
(TIF)

Figure S8 **Spatial distribution of bluefin tuna (** ***Thunnus thynnus*** **) larvae in relation to the geostrophic velocity gradient calculated at the characteristic scale (0.6 degrees).** Relative stage-1 larval abundances are shown in the maps such as dots.
(TIF)

Figure S9 **Spatial distribution of bullet tuna (** ***Auxis rochei rochei*** **) in relation to the salinity mean calculated at 0.75 degrees.** Relative stage-1 larval abundances are shown in the maps such as dots.
(TIF)

Figure S10 **Spatial distribution of bullet tuna (** ***Auxis rochei rochei*** **) in relation to the salinity gradient calculated at 0.75 degrees.** Relative stage-1 larval abundances are shown in the maps such as dots.
(TIF)
