# Supplementary figures and images for: Spatial Scale, Means and Gradients of Hydrographic Variables Define Pelagic Seascapes of Bluefin and Bullet Tuna Spawning Distribution
Source: PLoS One. 2014 Oct 27;9(10):e109338. doi: 10.1371/journal.pone.0109338 (PMC4210118; doi:10.1371/journal.pone.0109338)

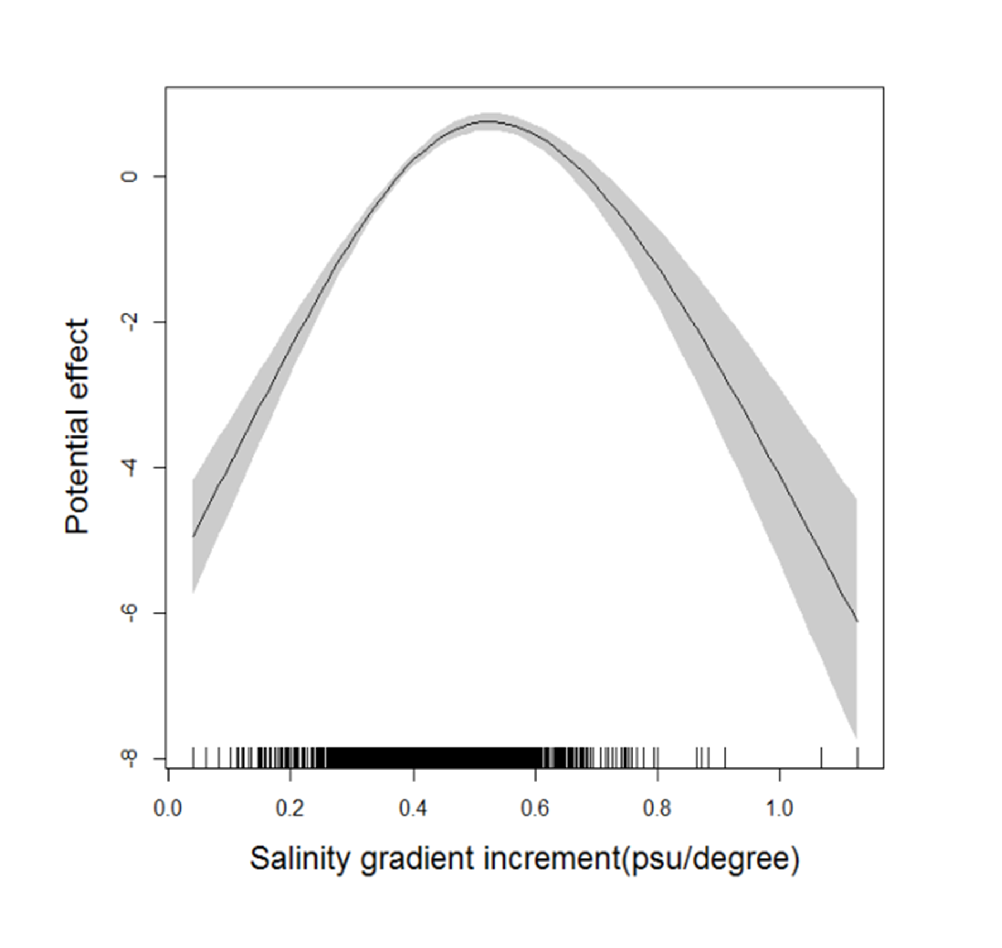

Supplement: Figure S1 — Model response of bluefin tuna in relation to salinity gradient processed at 0.75 degrees. Fitted line (solid line) and 95% confidence intervals (grey shaded areas) are shown. Whiskers on the x-axis show the locations of measurements. (TIF) [file pone.0109338.s001.tif]

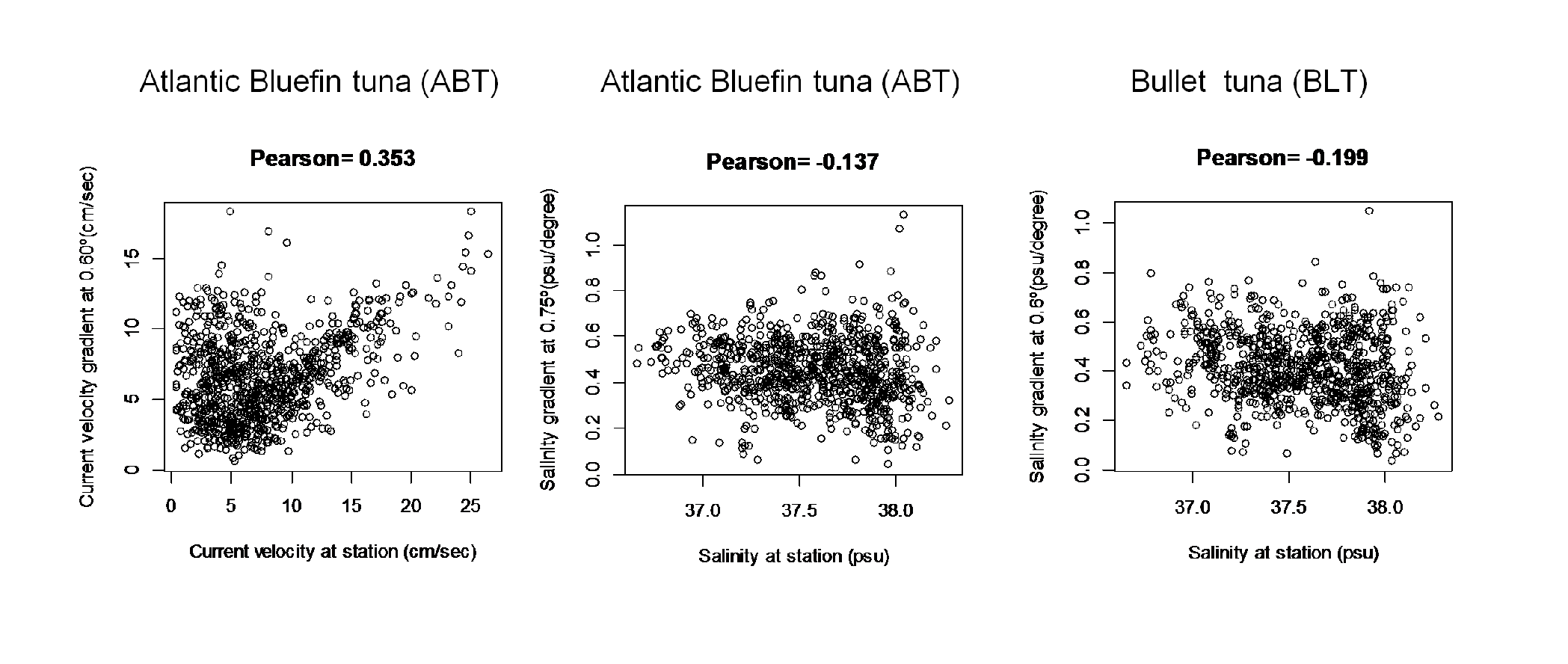

Supplement: Figure S2 — Correlation between the gradients at the characteristic scales and the hydrographical variables at the sampled station. A) Current velocity and B) salinity for Atlantic Bluefin tuna. C) Salinity for Bullet tuna. (TIF) [file pone.0109338.s002.tif]

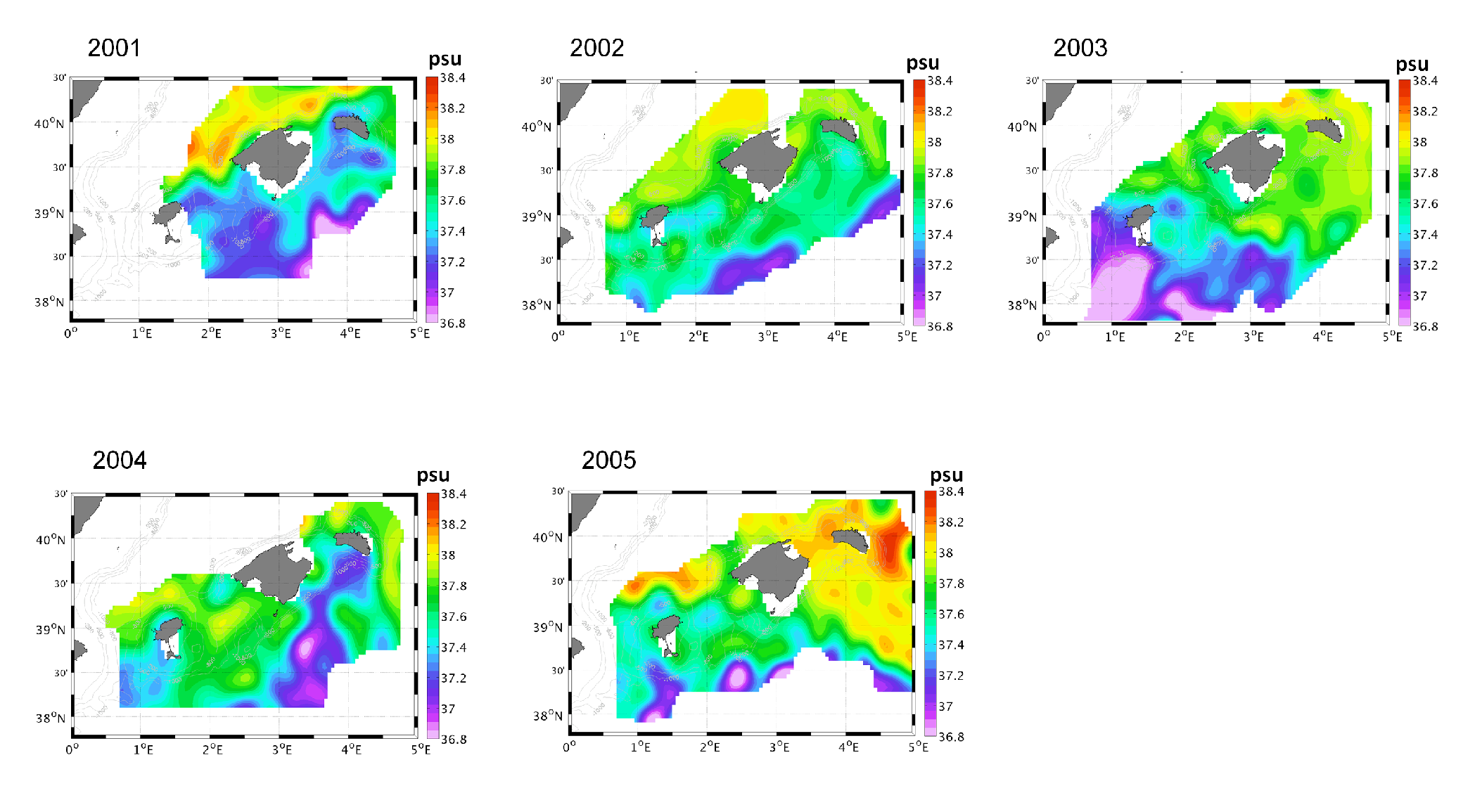

Supplement: Figure S3 — Sea surface salinities in the area during the five years analyzed (2001 to 2005). (TIF) [file pone.0109338.s003.tif]

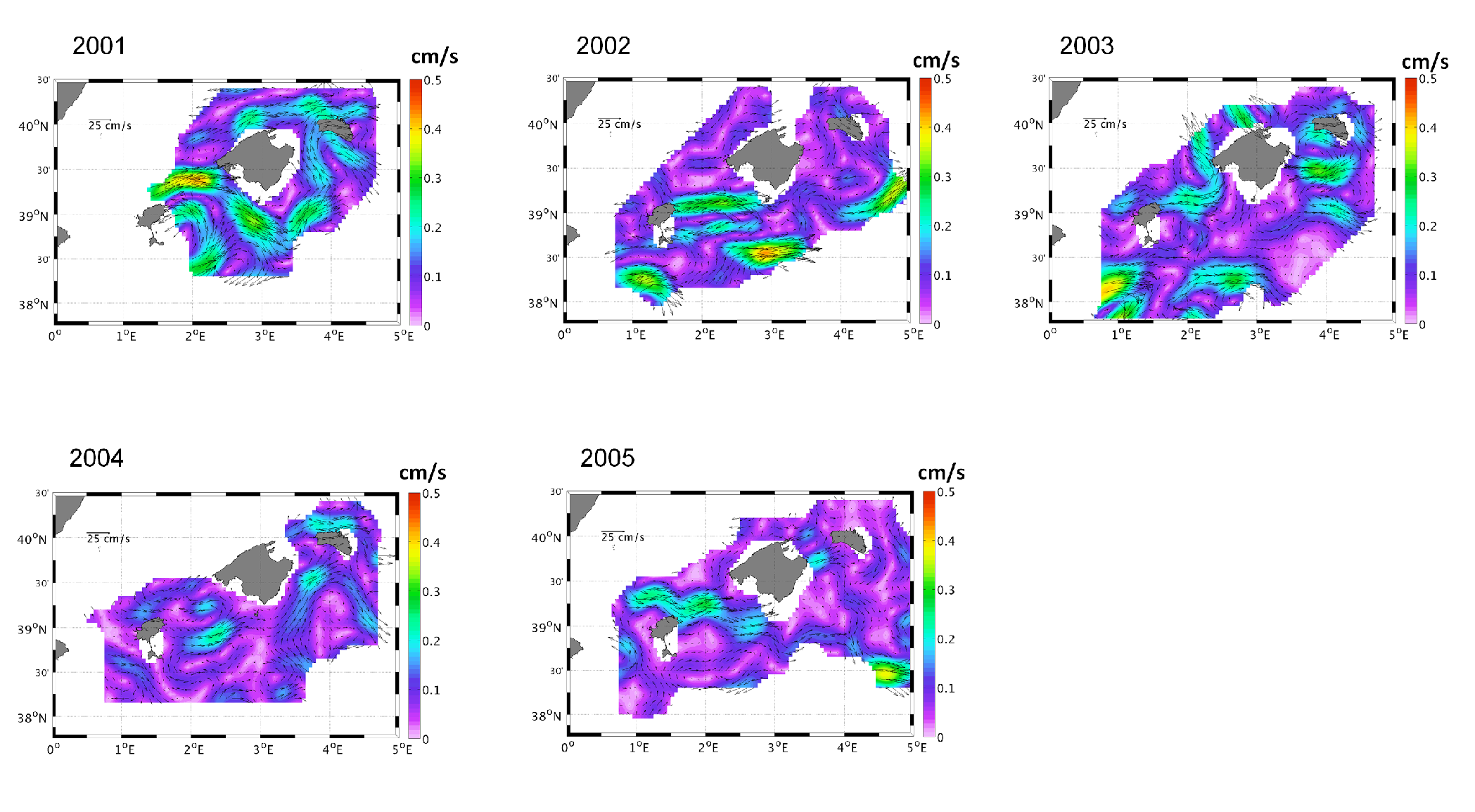

Supplement: Figure S4 — Sea surface geostrophic currents in the area during the five years analyzed (2001–2005). (TIF) [file pone.0109338.s004.tif]

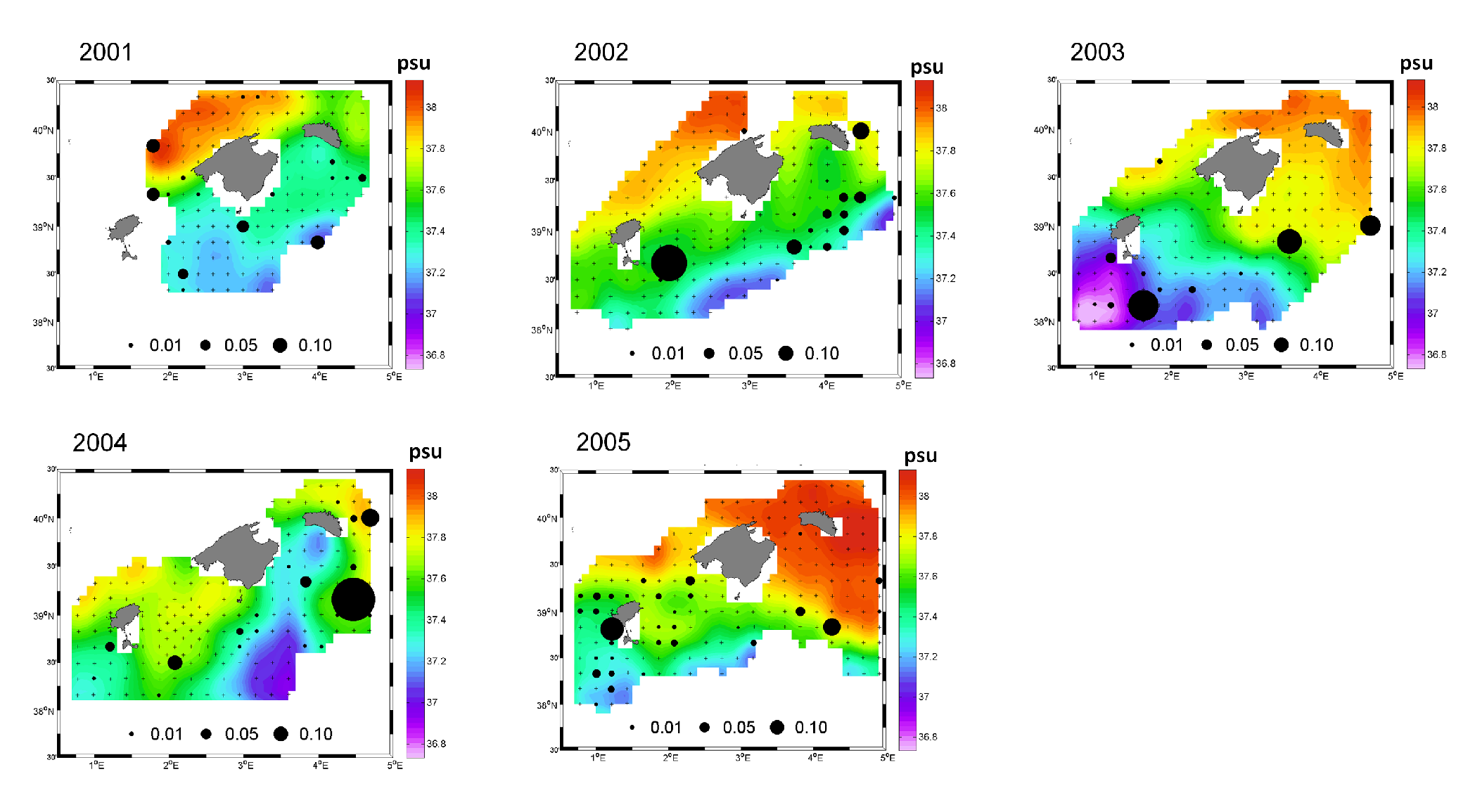

Supplement: Figure S5 — Spatial distribution of bluefin tuna ( Thunnus thynnus ) larvae in relation to the salinity mean calculated at its characteristic scale (0.6 degrees). Relative stage-1 larval abundances are shown in the maps such as dots. (TIF) [file pone.0109338.s005.tif]

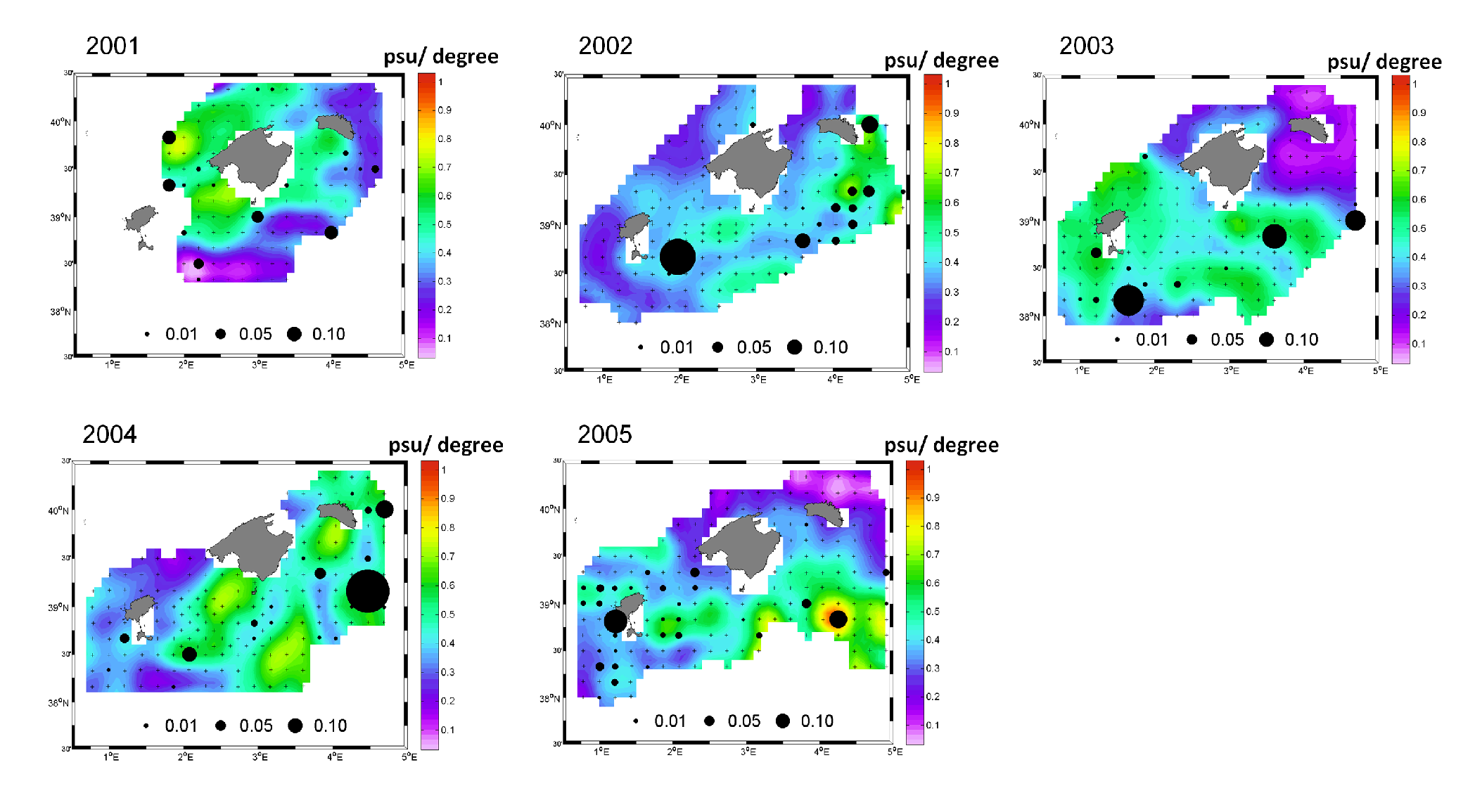

Supplement: Figure S6 — Spatial distribution of bluefin tuna ( Thunnus thynnus ) larvae in relation to the salinity gradient calculated at 0.6 degrees. Relative stage-1 larval abundances are shown in the maps such as dots. (TIF) [file pone.0109338.s006.tif]

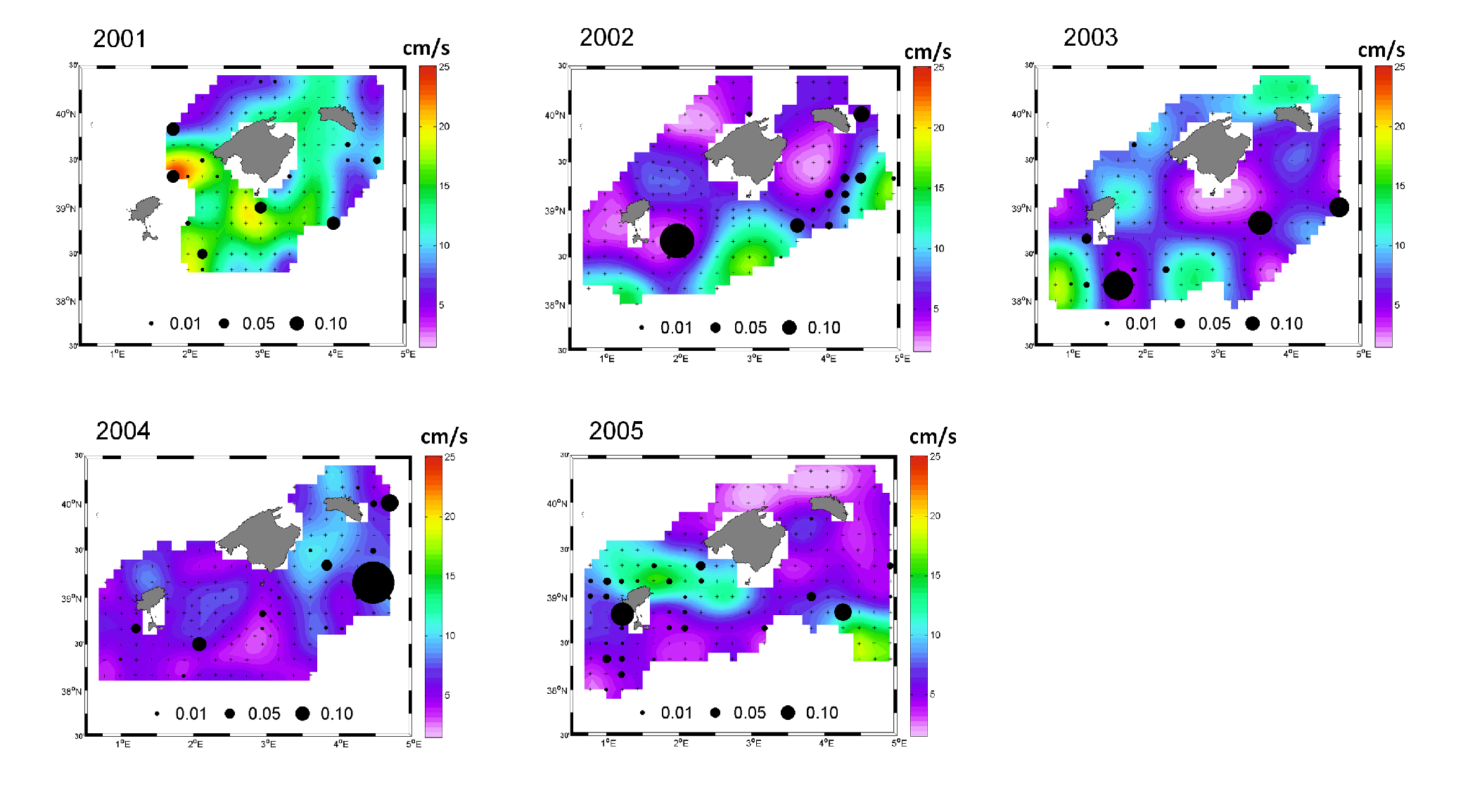

Supplement: Figure S7 — Spatial distribution of bluefin tuna ( Thunnus thynnus ) larvae in relation to the geostrophic velocity mean calculated at its characteristic scale (0.6 degrees). Relative stage-1 larval abundances are shown in the maps such as dots. (TIF) [file pone.0109338.s007.tif]

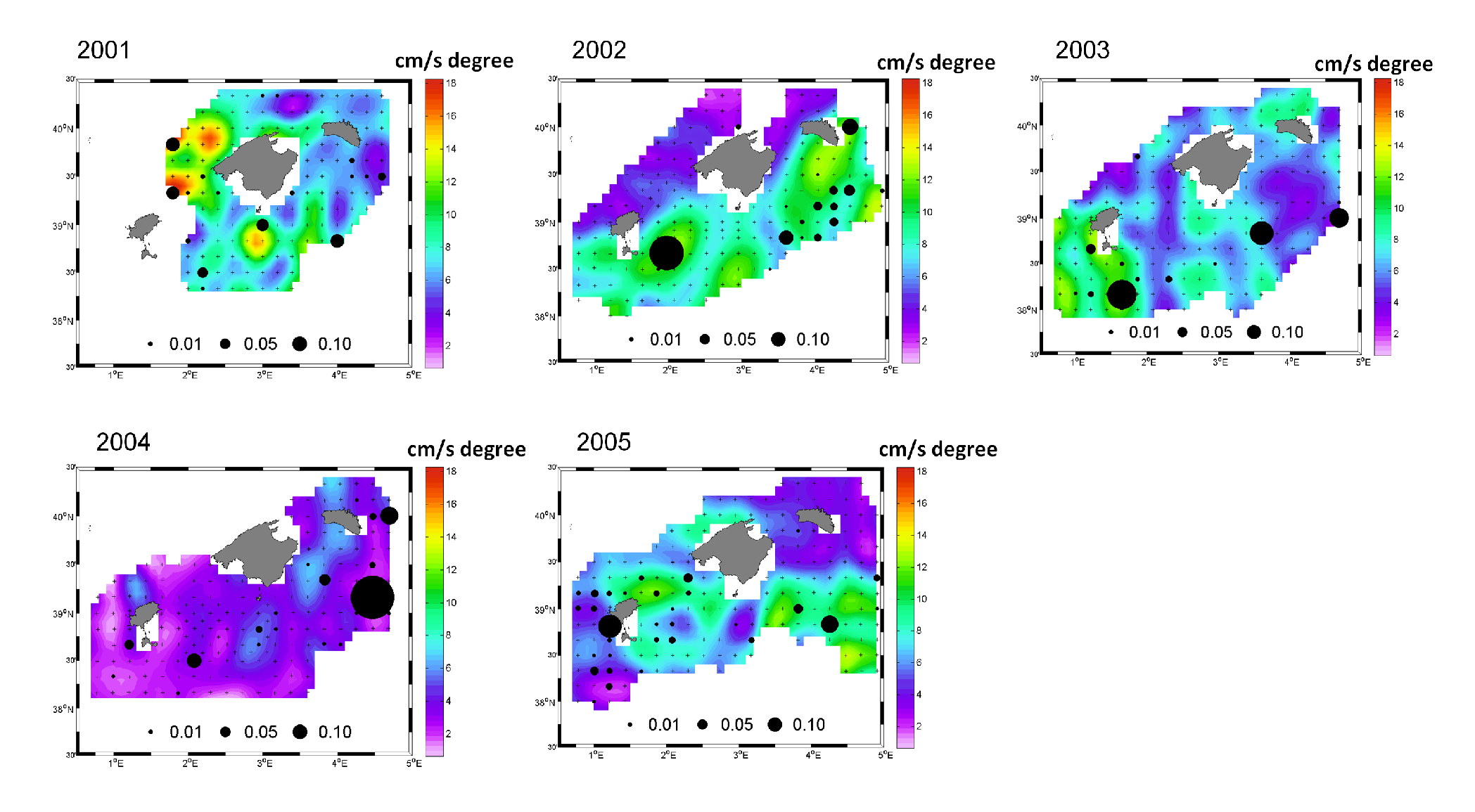

Supplement: Figure S8 — Spatial distribution of bluefin tuna ( Thunnus thynnus ) larvae in relation to the geostrophic velocity gradient calculated at the characteristic scale (0.6 degrees). Relative stage-1 larval abundances are shown in the maps such as dots. (TIF) [file pone.0109338.s008.tif]

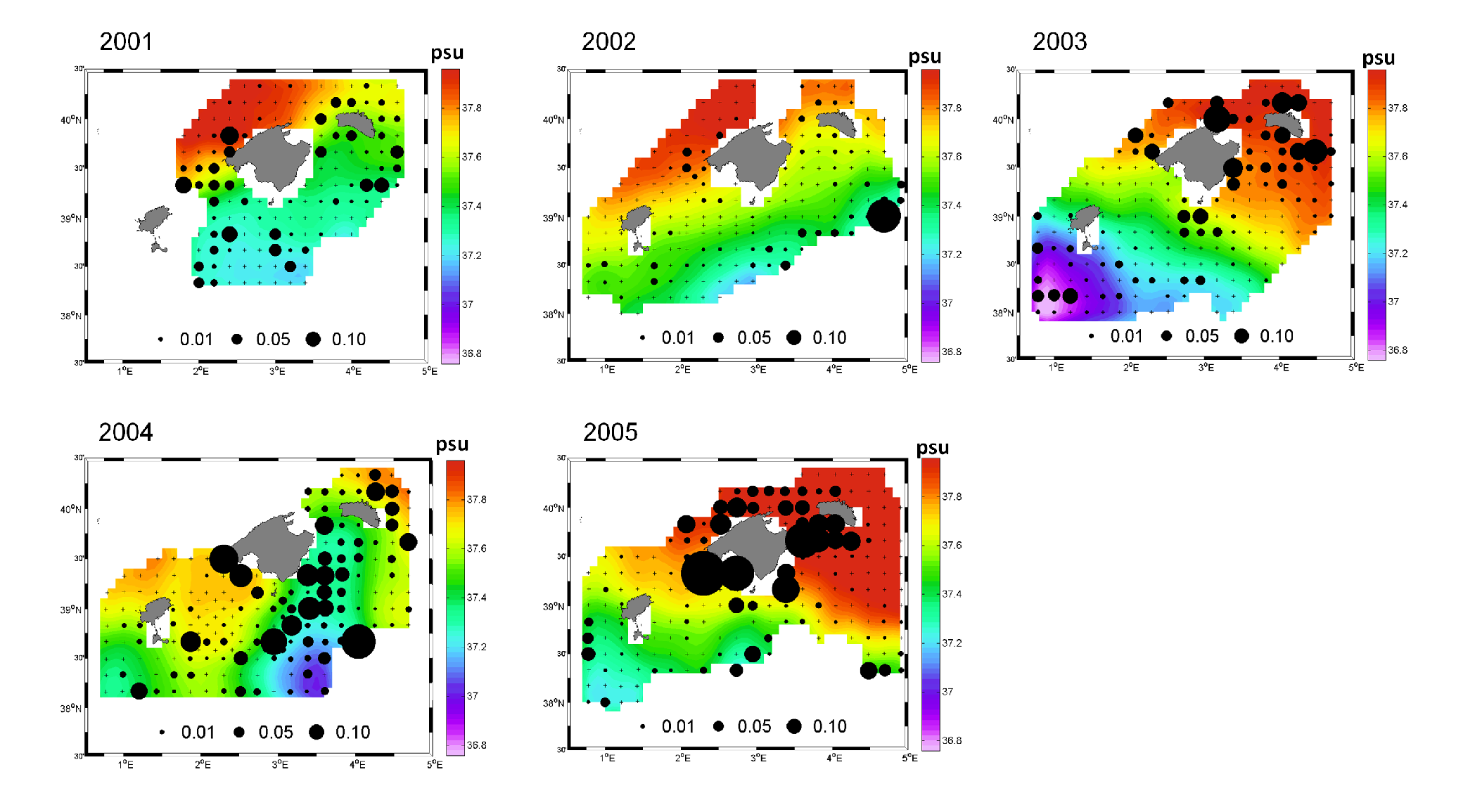

Supplement: Figure S9 — Spatial distribution of bullet tuna ( Auxis rochei rochei ) in relation to the salinity mean calculated at 0.75 degrees. Relative stage-1 larval abundances are shown in the maps such as dots. (TIF) [file pone.0109338.s009.tif]

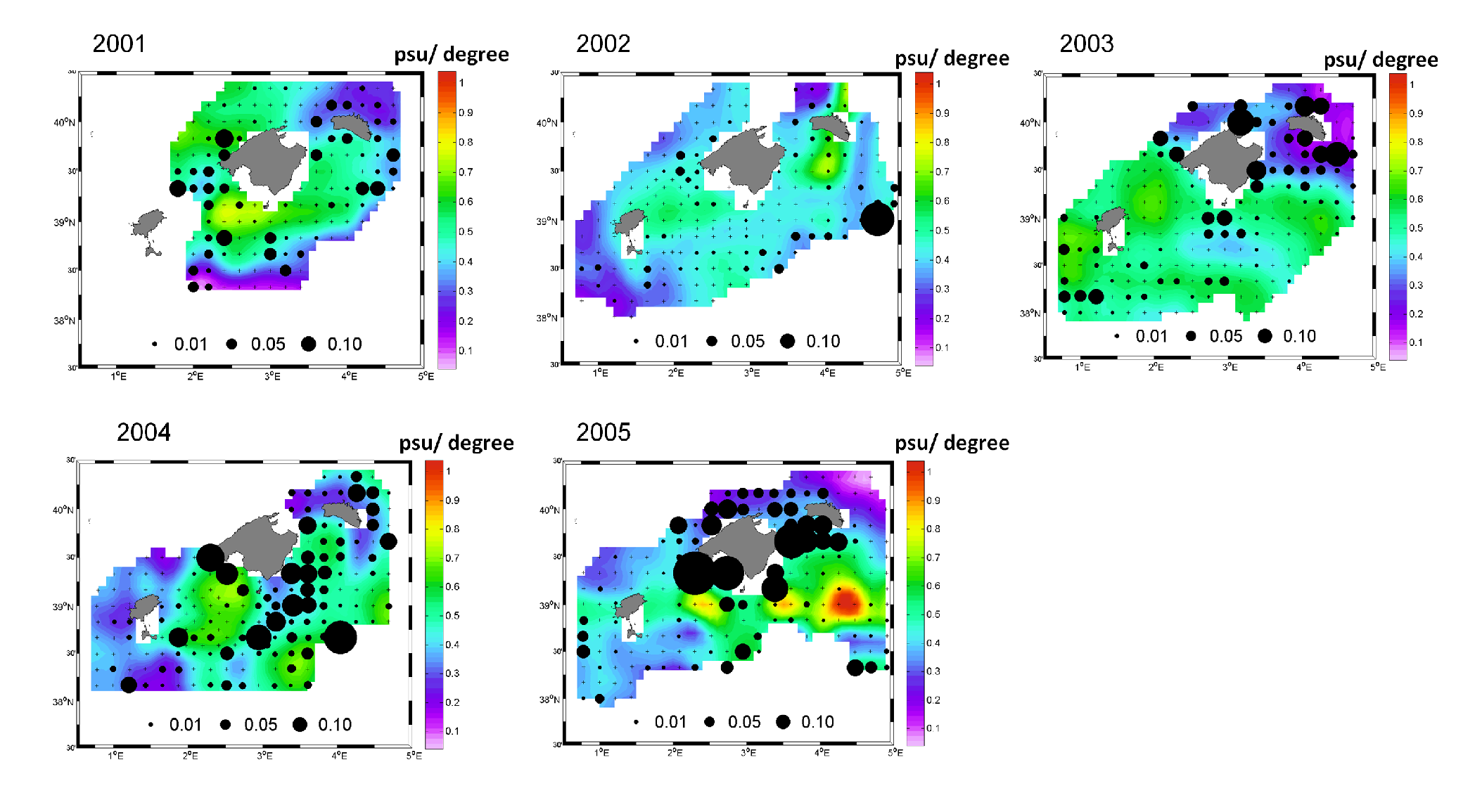

Supplement: Figure S10 — Spatial distribution of bullet tuna ( Auxis rochei rochei ) in relation to the salinity gradient calculated at 0.75 degrees. Relative stage-1 larval abundances are shown in the maps such as dots. (TIF) [file pone.0109338.s010.tif]
